# Radial Neck Osteotomy for Malunion of Radial Neck Fracture in Childhood

**DOI:** 10.1155/2015/871429

**Published:** 2015-08-09

**Authors:** Simon Vandergugten, Serge Troussel, Bernard Lefebvre

**Affiliations:** Department of Orthopaedic Surgery, Grand Hopital de Charleroi (GHdC), Grand'Rue 3, 6000 Charleroi, Belgium

## Abstract

In a case of a neglected radial neck fracture in childhood, the management of initial fracture and its complications are subjected to discussion. In children, open reduction should be avoided but an angulation less than 30° must be obtained. Several techniques exist to manage symptomatic malunion in adults, including resection, prosthesis, and osteotomy. When performing an osteotomy, it is important first to preserve an intact osseous hinge to avoid avascular necrosis and second to align the edge of the radial head articular surface with the lateral edge of the coronoid process, in order to avoid overstuffing elbow joint.

## 1. Introduction

Radial neck fracture management remains controversial in children and adults. As there is no consensus, the aim of this report is to discuss the different treatment options in adults suffering from a neglected radial neck fracture having occurred in childhood and resulting in a painful elbow stiffness.

## 2. Case Presentation

**Figure 1:**
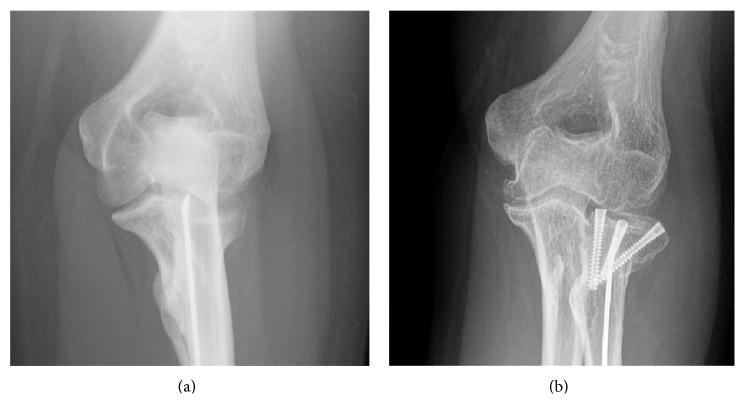
(a) Preoperative anteroposterior X-ray showing the radial neck malunion. (b) Postoperative anteroposterior X-ray showing the radial head fixation.

**Figure 2:**
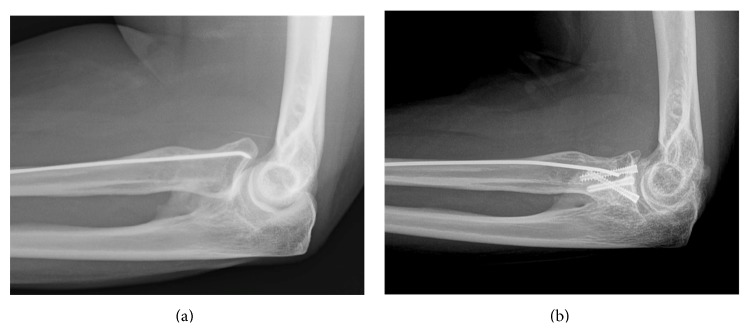
(a) Preoperative lateral X-ray showing the radial neck malunion of 60° dorsal angulation. (b) Postoperative X-ray showing the radial neck osteotomy and fixation.

**Figure 3:**
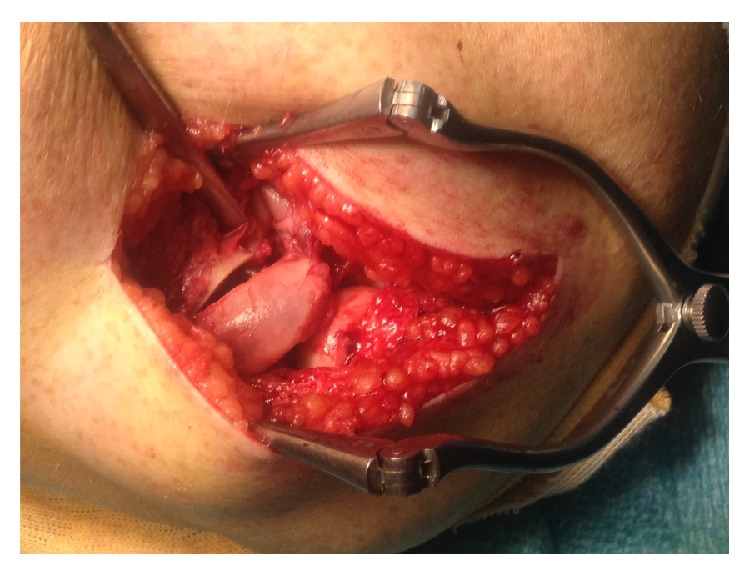
Radial neck palmar subtraction osteotomy of 60° on a pronated radius.

A woman aged 33 years has been consulting us for left lateral elbow pain for one year. At the age of 11, she suffered from an isolated radial neck fracture of Grade 4. She benefited at that time from a percutaneous reduction following Metaizeau technique. Early displacement occurred, leading to a second percutaneous Metaizeau procedure. Secondary displacement occurred again with an angulation of 60 to 70°. Apparent correct elbow ROM pushed the surgeon to not operate on her a third time. Medical records mentioned only a slight supination deficit. Clinical evolution was apparently good until the age of 32, when she started having progressive lateral elbow pain. Clinical examination objectified an elbow flexion at 125°, a 35° deficit extension, and 10-10° pronosupination. There was no elbow instability. Her pain was reproduced by writs extension against resistance. The Quick Disabilities of the Arm, Shoulder and Hand (Quick DASH) score was 65.9 (37.5 for work module). Conventional X-ray showed a radial neck malunion of 60° dorsal angulation on lateral elbow radiographs (Figures 1(a) and 2(a)). There was no chondrolysis on the CT-Scan, but a calcification at the level of the coronoid process of the ulna, which was not a synostosis, was notified. Neither the medical records nor the patient did report elbow dislocation at the time of the primitive traumatism, but the hypothesis of terrible triad elbow (posterior elbow dislocation, coronoid process fracture, and radial head fracture) could not be excluded. However, the elbow was stable at the time of examination and the problem clearly came from the radial neck. After physiotherapists' try for epicondylitis, we suggested that the patient takes a surgical option. The preoperative discussion was about radial head prosthesis, radial head excision, or radial neck osteotomy. Given her young age, we decided to do a radial neck osteotomy with a classical Kocher lateral approach and isolation of posterior interosseous nerve. Radial head cartilage was peroperatively intact, and we could objectify the conflict between anterior margin of the radial head and the coronoid fossa. We performed a radial neck palmar subtraction osteotomy of 60° on a pronated radius, with preservation of an osseous dorsal hinge (Figure 3). Given the delay since the first surgery, we were not able to remove the K-wire and were forced to cut it at the osteotomy site. To fix the radial head, we chose 3 intraosseous headless screws (Acutrak 2 Mini Headless Compression Screw System, Acumed LLC, Hillsboro, Oregon, USA) in order to give the maximum ROM possibility to the joint. Peroperatively passive 60°-60° pronosupination led us to leave the ulnar calcification. Elbow was immobilised in a cast for 3 weeks; then, the patient started physiotherapy to improve ROM. Postoperative radiographs showed radial head angulation of 0° and alignment of the radial head articular surface with the lateral edge of the coronoid process, with slight lateral translation of the radial head (Figures 1(b) and 2(b)). At 6 months, the elbow remained stable and ROM had improved with a complete flexion at 130°, a 15° deficit extension, and 60-60° pronosupination without impingement. Lateral elbow pain also improved as well as the Quick DASH score calculated at 4.5 (and 0 for work module).

## 3. Discussion

Management of pediatric radial neck fracture remains controversial [1]. There is no clear consensus about displacement angle acceptance, surgical indication, and the surgical technique to adopt. However, it is admitted that angulation beyond 30 degrees requires treatment and that close reduction techniques must be first tried [1–4]. In case of unstable or incomplete close reduction, percutaneous pinning following Metaizeau technique should be performed before open reduction [1, 2, 5]. As salvage procedure, neck osteotomy had been described in children in pain with neglected radial neck fracture [6]. In this case, if an open reduction had been performed after the second displacement in childhood, surgical management might have been avoided at the age of 33.

The most common approach of the radial head and neck is the lateral (posterior) approach of Kocher, passing between the anconeus muscle and the extensor carpi ulnaris muscle (ECU) exposing the proximal radius 3 to 6 cm before the crossing of the posterior interosseous nerve (PIN) as the forearm is, respectively, moved from supination to pronation [7, 8]. Annular ligament could then be incised anteriorly to the lateral ulnar collateral ligament (LUCL), which is the posterior part of the lateral collateral ligament, in order to avoid elbow instability [8].

In our case, there were three treatment possibilities. To improve the elbow ROM and hopefully ease the pain, we could simply have resected the radial head. The second possibility was to replace the radial head with metal prosthesis in correct position [9]. The third possibility was to keep the patient's radial head and perform a radial neck correction osteotomy. Regarding the initial treatment of comminuted radial head fracture, it has been proven that internal fixation or radial head replacement had better clinical and functional outcomes than resection [10–12]. This case was different but we straightaway excluded simple resection. The decision between prosthesis and osteotomy was made peroperatively. Radial head cartilage was intact; there was thus absolutely no reason to resect it. Given the 60° dorsal angulation of radial head articular surface, we decided to perform a palmar subtraction osteotomy of 30° in order to obtain an articular surface perpendicular to the radial shaft axis. In order to move away PIN, osteotomy was done on a pronated radius [7].

Anatomical studies have proven that the blood supply to the radial head is tenuous and mainly periosseous, coming from a pericervical ring around the radial neck, resulting from the union of branches of radial recurrent artery laterally, periosseous branches of ulnar artery medially, and interosseous recurrent artery posteriorly [13, 14]. That is why it is important to preserve an osseous and periosseous hinge in order to avoid avascular necrosis of the radial head.

To assess the correct height of the radial head articular surface, we depended on reference points for radial head prosthesis size. To avoid overstuffing the elbow joint, CT-Scan study suggested just to align the edge of the radial head articular surface with the lateral edge of the coronoid process articular surface which is easily seen with the used surgical approach [15].

Several methods exist to fix the radial head, as in the case of radial neck fracture: crossed K-wires, antegrade interfragmentary screw, and anatomical T-plate with compression or locked screws. Biomechanical studies may attest an advantage of the interfragmentary screw in terms of rigidity in bending and torsion [16], whereas a recent review brings similar results of different types of fixation devices [12]. It has been proven that plate fixation leads to higher destruction of periosseous vascularisation than screw [13]. In addition, we thought that a T-plate could increase the elbow stiffness and would necessitate an additional removing surgery.
